# *FLEXR-MSA*: electron-density map comparisons of sequence-diverse structures

**DOI:** 10.1107/S2052252525001332

**Published:** 2025-02-27

**Authors:** Timothy R. Stachowski, Marcus Fischer

**Affiliations:** a Department of Chemical Biology and Therapeutics, MS 1000 St Jude Children’s Research Hospital 262 Danny Thomas Place Memphis TN38105 USA; University of Michigan, USA

**Keywords:** electron-density sampling, protein conformational landscape, HSP90, isoforms, dynamics, ligand discovery

## Abstract

Proteins with near-identical sequences often share similar static structures. Yet, comparing crystal structures is limited or even biased by what has been included or omitted in the deposited model. Information about unique dynamics is often hidden in electron-density maps. Currently, automatic map comparisons are limited to sequence-identical structures. To overcome this limitation, we developed *FLEXR-MSA*, which enables unbiased electron-density map comparisons of sequence-diverse structures by coupling multiple sequence alignment (MSA) with electron-density sampling. *FLEXR-MSA* generates visualizations that pinpoint low-occupancy features on the residue level and chart them across the protein surface to reveal global changes. To exemplify the utility of this tool, we probed electron densities for protein-wide alternative conformations of HSP90 across four human isoforms and other homologs. Our analysis demonstrates that *FLEXR-MSA* can reveal hidden differences among HSP90 variants bound to clinically important ligands. Integrating this new functionality into the *FLEXR* suite of tools links the comparison of conformational landscapes hidden in electron-density maps to the building of multi-conformer models that reveal structural/functional differences that might be of interest when designing selective ligands.

## Abstract 2

*FLEXR-MSA* extends the *FLEXR* suite of tools by enabling electron-density map comparisons of sequence-diverse proteins.

## 1. Introduction

Proteins are peripatetic (Matthews, 2010 ▸), so that at each point in time they exist as a collection of major and minor states. In response to perturbations such as ligand binding (Merski *et al.*, 2015 ▸; Wankowicz *et al.*, 2022 ▸; Stachowski & Fischer, 2022 ▸) or temperature (Fischer, 2021 ▸; Fischer *et al.*, 2015 ▸; Keedy, 2019 ▸; Stachowski *et al.*, 2022 ▸) the relative populations of these states are reshaped (Frauenfelder *et al.*, 1991 ▸; Henzler-Wildman & Kern, 2007 ▸; Yabukarski, Doukov, Mokhtari *et al.*, 2022 ▸). This flexibility is essential to many functions, including enzyme catalysis and membrane transport. Detecting areas of flexibility can reveal new opportunities for developing biological or technical advances (Bradford *et al.*, 2021 ▸; Fischer *et al.*, 2014 ▸; Aplin *et al.*, 2022 ▸; Yabukarski, Doukov, Pinney *et al.*, 2022 ▸).

Despite the recent ‘resolution revolution’ in cryo-electron microscopy (Kühlbrandt, 2014 ▸), X-ray crystallography is still the most popular tool for determining near-atomic resolution protein structures. Crystallographic electron-density maps solved to sufficient resolution contain information about dynamics such as weakly populated and high-energy minor states (Fraser *et al.*, 2011 ▸; Lang *et al.*, 2010 ▸; Pearce *et al.*, 2017 ▸; Pearce & Gros, 2021 ▸). Estimates from retrospective analyses of deposited X-ray data suggest that up to a third of protein side chains show evidence of minor states in electron-density maps but are not accounted for in the corresponding models (Fraser *et al.*, 2011 ▸; Bradford *et al.*, 2021 ▸; Shapovalov & Dunbrack, 2007 ▸; Lang *et al.*, 2010 ▸). The incompleteness of published models is partially due to the fact that the signal for flexible features such as alternative conformations of side chains is often weak. This makes it challenging to accurately discern genuine signal from experimental noise. Additionally, incoporating conformational dynamics into models necessitates manual intervention, which is cumbersome and inaccessible for non-crystallographers. Emerging automated multi-state modeling tools such as *qFit*, *Phenix-MD* and *FLEXR*try to bridge this gap (Riley *et al.*, 2021 ▸; Burnley *et al.*, 2012 ▸; Stachowski & Fischer, 2023 ▸, 2024 ▸). Electron-density measurements without explicit model building using tools such as *Ringer* (Lang *et al.*, 2010 ▸) circumvent these pitfalls and allow the visualization of side-chain dynamics without modeling bias. Current wisdom supports that alternate side chains can be confidently interpreted in weak electron density (≳0.3σ; Lang *et al.*, 2010 ▸), which enables older maps deposited at a time of more cautious modeling guidelines (previously >1σ) to be searched. While ensemble methods build comprehensive models, comparing structural differences between proteins with non-identical sequences remains challenging.

One of the cornerstone approaches for probing the protein conformational landscape is through mutagenesis (Winter *et al.*, 1982 ▸), where structural and functional consequences are monitored when substituting amino acids with different properties (Fowler & Fields, 2014 ▸). Nature took advantage of this to develop highly specialized proteins from related ones, for example through sequence divergence (Chothia & Lesk, 1986 ▸) or alternative splicing (Baralle & Giudice, 2017 ▸). However, nature’s ingenuity creates a large hurdle for drug discovery. Poor selectivity of sequence-related but functionally distinct proteins often leads to serious off-target effects for clinical targets such as human histone deacetylase (Ma *et al.*, 2016 ▸), carbonic anhydrase (Alterio *et al.*, 2012 ▸), kinases (Ferguson & Gray, 2018 ▸) and bromodomains (Liu *et al.*, 2017 ▸). Generally, aspects of protein flexibility can be used to improve ligand affinity and selectivity (Teague, 2003 ▸).

Another well known example is the heat-shock protein 90 (HSP90) family of molecular chaperones. HSP90 proteins drive all ten hallmarks of cancer (Hanahan & Weinberg, 2011 ▸; Garg *et al.*, 2016 ▸) but no inhibitor has been clinically approved outside Japan (Yuno *et al.*, 2018 ▸). Humans possess four HSP90 isoforms (Hsp90α, Hsp90β, Grp94 and Trap1) that share greater than 90% sequence identity in the N-terminal domain (NTD) binding site alone, where Hsp90α and Hsp90β differ by only two residues (Stachowski *et al.*, 2023 ▸; Supplementary Fig. S1). An isoform-selective inhibitor is a promising avenue to avoid inducing the cellular heat-shock response and eventual tumor resistance (Mishra *et al.*, 2021 ▸; Huck *et al.*, 2019 ▸; Ernst *et al.*, 2014 ▸; Gewirth, 2016 ▸). Likewise, Hsp90α is targeted in antifungal drug development, but the close similarity between the human and fungal homologs causes severe host toxicities (Cowen *et al.*, 2009 ▸; Supplementary Fig. S2). *Candida albicans* is the most common fungal pathogen affecting humans. While *C. albicans* Hsp90α shares 72% sequence identity with the human homolog NTD, the binding site remains largely conserved with only two residues changing: S52A and V186L (according to the human sequence numbering; Supplementary Figs. S1 and S2). Despite their similar sequences, there are major structural differences in ligand binding between the *C. albicans* and human homologs that might open routes for developing targeted antifungal therapies (Whitesell *et al.*, 2019 ▸). These differences primarily include rearrangements in the ATP lid-loop region, which is known to be highly dynamic and ligand-responsive in the human form (Amaral *et al.*, 2017 ▸; Stachowski & Fischer, 2022 ▸) but possibly more so in *C. albicans* (Whitesell *et al.*, 2019 ▸). With HSP90 proteins being remarkly flexible (Stachowski & Fischer, 2022 ▸) and the human isoforms exhibiting subtle but meaningful structural differences, this opens new routes for selective inhibition (Khandelwal *et al.*, 2018 ▸; Huck *et al.*, 2019 ▸; Stachowski *et al.*, 2023 ▸).

Here, we combine electron-density map sampling with multiple sequence alignment (MSA) into *FLEXR-MSA* as a tool for comparing the electron densities of structures with mutations, dissimilar sequences and misnumbered residues. For HSP90, this tool enabled us to directly probe electron-density maps for protein-wide alternative side-chain conformations across three homologs. More generally, our analysis demonstrates that *FLEXR-MSA* can offer new insights into structural differences among sequence-dissimilar proteins that are often missed in static models. The tool is open source and is available within *FLEXR* on GitHub at https://github.com/TheFischerLab/FLEXR.

## 2. Materials and methods

Coordinates and structure factors (Supplementary Tables S1 and S2) were taken from the Protein Data Bank (PDB; Berman *et al.*, 2000 ▸). For Hsp90α, we compared structures according to Whitesell and coworkers except in the case of the apo human structure (Supplementary Table S1; Whitesell *et al.*, 2019 ▸), where the authors used PDB entry 1yer, which was deposited without structure factors. We used PDB entry 1uyl, which is also apo, solved at a comparable resolution (1.7 Å for PDB entry 1yer and 1.4 Å for PDB entry 1uyl) and has the same lid conformation (‘in’). Maps were examined with *Ringer* as described previously (Lang *et al.*, 2010 ▸). *PyMOL* (Schrödinger, New York, USA) was used to generate images and to detect conformational changes in the ATP lid. All-atom r.m.s.d. values and structural superpositions were also performed in *PyMOL* using align mobile.pdb, target.pdb, cycles=0. These structure-based alignments are not considered in *FLEXR-MSA*. Chains in structures with multiple copies were treated as separate models, except in the case of PDB entry 3opd where, due to the lower resolution (2.6 Å), only the *A* chain was considered. Binding-site volumes and hydrophiblic–hydrophobic balance were calculated with *SiteMap* (Halgren, 2009 ▸) in *Maestro* (Schrödinger, New York, USA).

*FLEXR-MSA* was written in Python 3.9 and packaged within the *FLEXR* suite of tools. Full functionality of *FLEXR*, including the GUI (Stachowski & Fischer, 2024 ▸), requires *Coot* 1.1.10 (Emsley, 2023 ▸), which we recommend installing through *CCP4* version 9 (Agirre *et al.*, 2023 ▸). *FLEXR* is available as an open-source program in a GitHub repository (https://github.com/TheFischerLab/FLEXR) and requires the Biopython, Matplotlib, Numpy, Pandas and SciPy Python packages. *Ringer* is available in the *mmtbx* library (https://cctbx.github.io/mmtbx/mmtbx.html) or in *Phenix* (Liebschner *et al.*, 2019 ▸). *MUSCLE* version 5.2 (Edgar, 2004 ▸) is also available through Homebrew (https://github.com/brewsci/homebrew-bio/blob/develop/Formula/muscle.rb) or can be installed separately (https://www.drive5.com/muscle). *Ringer* peak detection and peak subtraction were performed as described previously (Stachowski *et al.*, 2022 ▸). Pearson correlation coefficient (CC) calculations were performed with the SciPy Python package (Virtanen *et al.*, 2020 ▸). Surface visualizations require *PyMOL*. A detailed protocol for running *FLEXR-MSA* is given in the Supplementary Methods.

## 3. Results

### 3.1. Program description

**Figure 1:**
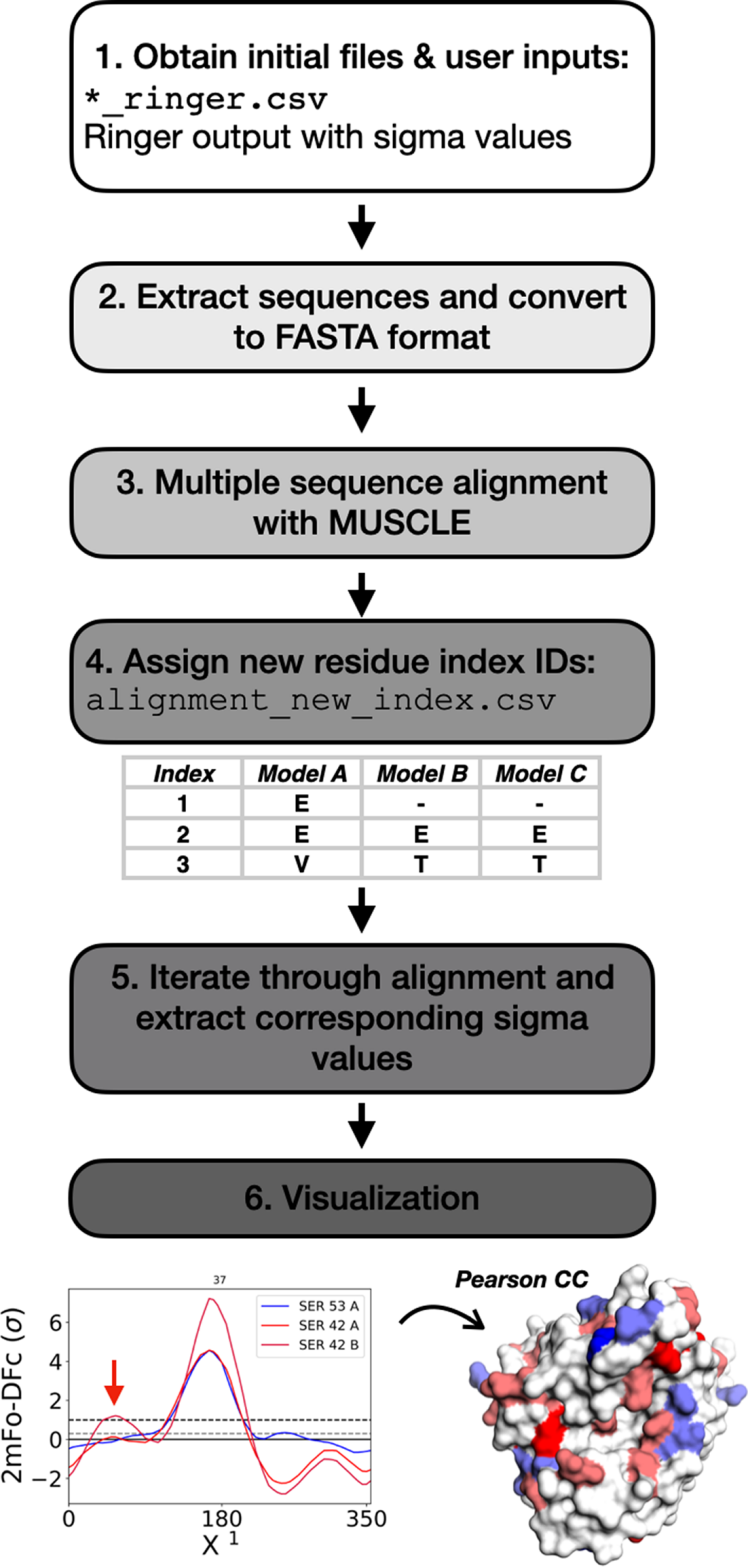
*FLEXR-MSA* workflow for comparison of alternative side-chain conformations in electron-density maps across related, sequence-diverse proteins. (1) *FLEXR-MSA* reads in the CSV output file with the σ values from *Ringer* (Lang *et al.*, 2010 ▸), (2) extracts the amino-acid sequence from the *Ringer* output, formats the sequences into *FASTA*, and (3) performs a multiple sequence alignment (MSA) using *MUSCLE* (Edgar, 2004 ▸). (4) Residues in each sequence are re-indexed according to their position in the alignment. (5) σ values at each position in the alignment for each sequence are extracted. (6) σ values are plotted as classical *Ringer* plots where the plot title corresponds to the MSA index and the residue numbers are shown in the legend. A PDB file is also generated that contains median Pearson CC values that can be visualized in *PyMOL*.

The *FLEXR-MSA* workflow is illustrated in Fig. 1 ▸. After the user runs *Ringer*, *FLEXR-MSA* starts from the standard *Ringer* CSV output files that contain σ measurements taken around each dihedral angle (χ) for each amino-acid residue, except Gly and Ala, in a PDB structure (Lang *et al.*, 2010 ▸). The amino-acid sequence is extracted from the *Ringer* output and organized into FASTA format. A multiple sequence alignment (MSA) is performed with *MUSCLE* (Edgar, 2004 ▸). Residues are renumbered according to their location in the MSA, and their relation to the numbering in the input *Ringer* CSVs are saved in a look-up table. To produce classical ‘*Ringer* plots’ (σ values as a function of side-chain rotation angle) for each residue, σ values are extracted at each position in the alignment for each sequence. These image files are saved to the working directory; the plot title and file name correspond to the MSA position. This process is repeated for each χ angle. To facilitate quick cross-comparison the original PDB residue number and chain ID is captured in the figure legend (see Fig. 1 ▸). The alignment files are also saved and can be manually adjusted and reloaded. Colors can be defined by the user and otherwise are automatically assigned (see Supplementary Methods). Median Pearson CC values are calculated and saved in the *B*-factor column of a given PDB file to be visualized in *PyMOL*. Starting from the *Ringer* output, the whole process takes less than a minute for these HSP90 comparisons.

### 3.2. Detecting alternative conformations across isoforms

To illustrate the utility of *FLEXR-MSA*, we chose the structurally dynamic HSP90 family of molecular chaperones. The high sequence identity and structural similarity among its four human isoforms has made it difficult to discover isoform-selective compounds. We applied *FLEXR-MSA* to structures of each isoform bound to the same fragment, *N*,*N*-dimethyl-7H-purin-6-amine (6DMP; PDB ID 42C; Stachowski *et al.*, 2023 ▸). 6DMP contains the core purine scaffold that is present in the native substrate ATP and is a common starting point in ligand discovery.

**Figure 2:**
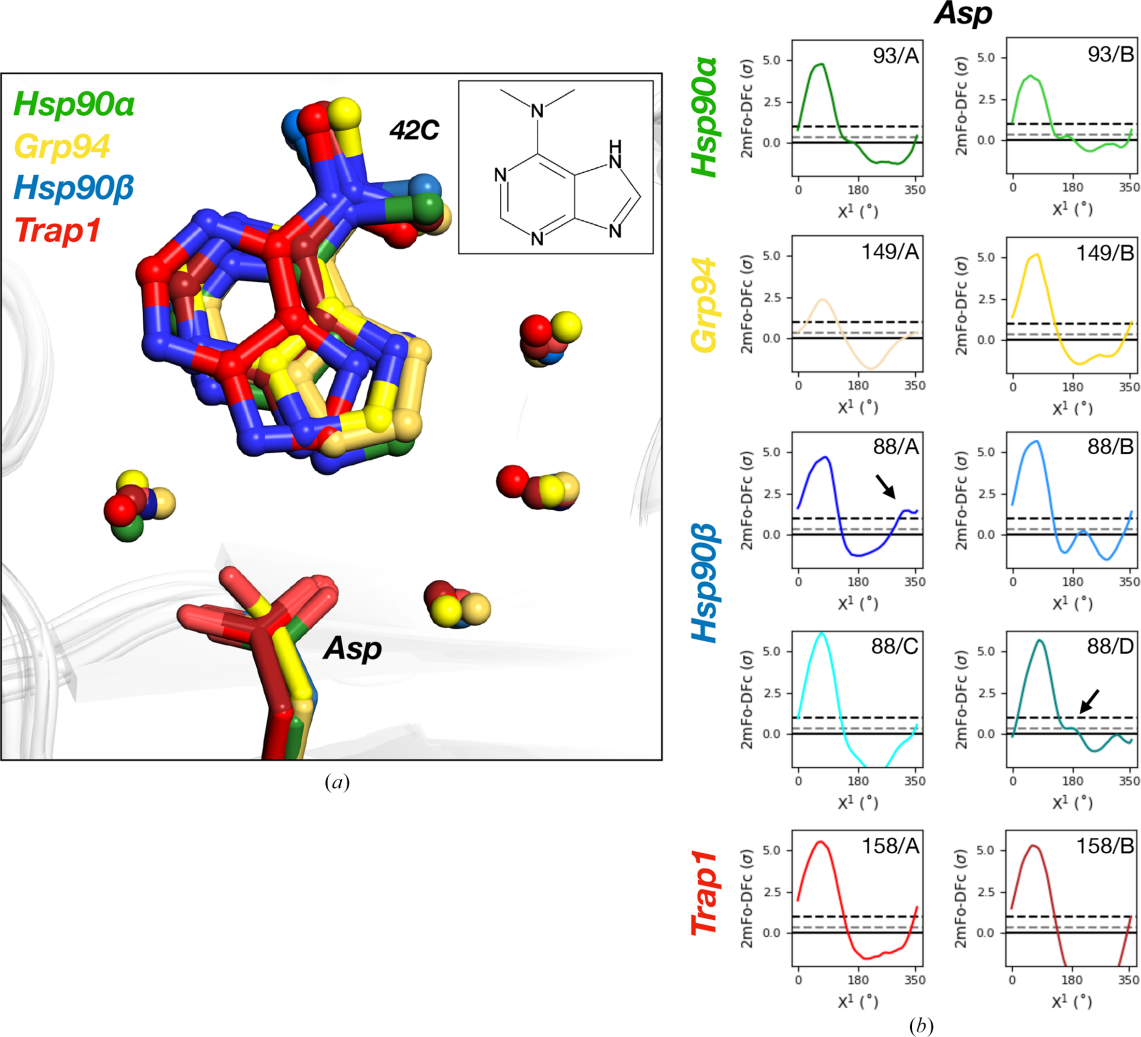
*FLEXR-MSA* reveals previously unnoticed isoform-specific conformations across all four 6DMP-bound human HSP90s. (*a*) Hsp90α (green), Hsp90β (blue), Grp94 (yellow) and Trap1 (red) bound to 6DMP (inset, PDB ligand ID 42C). (*b*) *Ringer* plot produced by *FLEXR-MSA* showing additional conformations (arrows) of a conserved binding-site Asp in Hsp90β chains *A* (∼340°) and *D* (∼190°). The solid line corresponds to 0σ. The gray dashed line corresponds to 0.3σ, the *Ringer* cutoff for modeling. The black dashed line corresponds to 1σ, the conventional modeling threshold.

To find changes that may impact ligand binding, we focused on binding-site residues. All isoforms contain a conserved Asp that is often exploited to hydrogen-bond to ligands (Chiosis *et al.*, 2001 ▸). This Asp is surrounded by a conserved water network that varies in position due to the loss of a hydrogen bond from a nearby mutation from Ser in Hsp90α to Ala in Hsp90β, Grp94 and Trap1. This distinguishing feature was previously exploited to design ligands that displace or retain certain waters and improve α/β selectivity (Khandelwal *et al.*, 2018 ▸; Mishra *et al.*, 2021 ▸; Huck *et al.*, 2019 ▸). Here, all isoforms share the same predominate conformation of the Asp [Fig. 2 ▸(*a*)]. However, *FLEXR-MSA* reveals that two of the four chains in Hsp90β contain an additional Asp rotamer that is not present in the other isoforms (*A* at ∼340° and *D* at ∼190°) [Fig. 2 ▸(*b*)]. It is conceivable that the additional conformations may be facilitated by the greater flexibility of the water network in Hsp90β over Hsp90α due to the Ser-to-Ala mutation that differentiates the two cytoplasmic isoforms.

### 3.3. Detecting specific conformations between human and *C. albicans* Hsp90α

To better understand homolog-specific flexibility in ligand binding, we used *FLEXR-MSA* to reanalyze four pairs of human and *C. albicans* Hsp90α structures: one apo and three bound to matching ligands first reported by Whitesell *et al.* (2019 ▸) (Supplementary Table S2).

**Figure 3:**
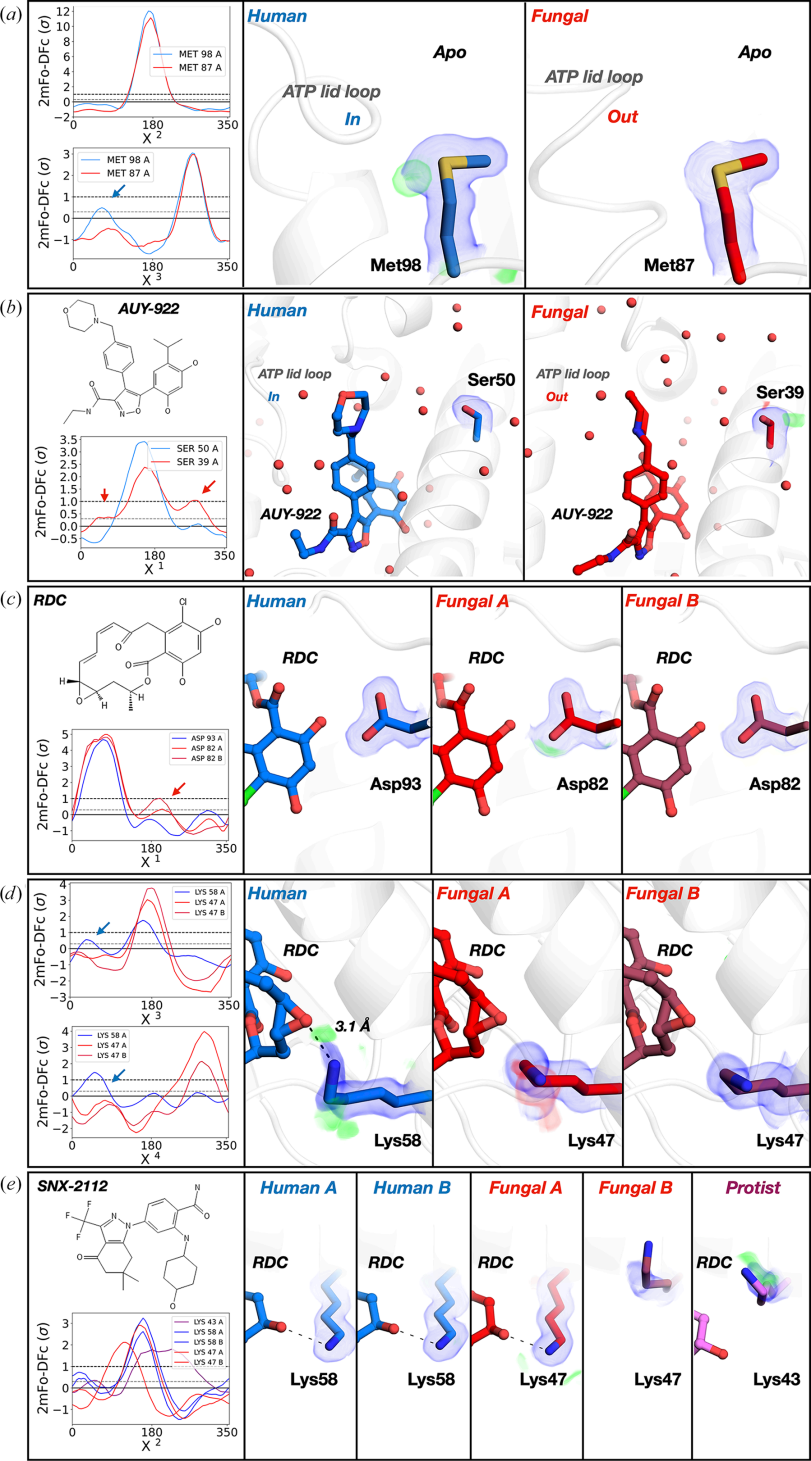
Homolog-specific HSP90 conformations. (*a*) Comparison of human (blue) and *C. albicans* (red) apo Hsp90α shows an additional conformation of a binding-site Met in the *Ringer* plot for the human protein. (*b*) Comparison of human and *C. albicans* Hsp90α bound to AUY-922 shows an additional conformation of a Ser in the *C. albicans* protein in the inset *Ringer* plot. (*c*) Radicicol (RDC) bound to *C. albicans* Hsp90α has an additional conformation of a conserved Asp in the *C. albicans* form, while Lys58/47 is more variable in the human form (*d*, *e*). (*e*) Comparison of SNX-2112 bound to human, *C. albicans* and *T. brucei* (purple) Hsp90α. The *A *and *B* chains are shown for human and *C. albicans* and chain *A* is shown for *T. brucei*. The inset *Ringer* plot shows that the Lys is conformationally variable between homologs bound to the conformation-responsive SNX-2112 ligand. Dotted lines represent hydrogen bonds detected with *PyMOL*. 2*F*_o_ − *F*_c_ maps are shown in blue and contoured at 1σ. *F*_o_ − *F*_c_ maps are shown in red and green and contoured at ±2σ.

First, we inspected the binding site in apo *C. albicans*and human structures. *FLEXR-MSA* revealed that the apo electron-density map for human Hsp90α shows an alternative conformation of a conserved methionine that is not present in the *C. albicans* map [Fig. 3 ▸(*a*)]. The origin of this change in the population of Met98/87 (human/*C. albicans* numbering) conformations might be a consequence of the different position of the lid, which is in the ‘in’ conformation in the human protein and the ‘out’ conformation in that from *C. albicans* (r.m.s.d. of 1.5 Å; Corbett & Berger, 2010 ▸). The alternative conformation repositions the terminal sulfur–carbon group of Met98/87. As a consequence of these conformational differences the binding site shifts its hydrophilic–hydrophobic balance towards more hydrophobic (0.71 in human and 0.47 in *C. albicans*). This change provides different surfaces to target, although the binding-site volume change may appear to be negligible (280 Å^3^ in the human protein versus 277 Å^3^ in that from *C. albicans*).

Secondly, we were interested in understanding the impact of sequence differences on binding AUY-922 (luminespib), which is an experimental drug candidate that reached Phase II (Felip *et al.*, 2018 ▸) in clinical trials for several cancer types. Binding of AUY-922 leads to different protein and ligand conformations between human and *C. albicans* Hsp90α. The lid in the human–AUY-922 complex is in the ‘in’ state, mirroring the apo conformation, while the lid in *C. albicans* is in the ‘helical’ state (lid r.m.s.d. of 3.6 Å; Whitesell *et al.*, 2019 ▸; Supplementary Fig. S3). This change in lid state repositions the terminal morpholine substituent and leads to different polar interactions, with an overall ligand r.m.s.d. of 1.4 Å. Differences in ligand position cascade throughout the binding site and reposition water molecules and proximal unengaged residues such as Ser50/39, which has an additional conformation in *C. albicans* [Fig. 3 ▸(*b*)]. In the newly identified conformation, the Ser hydroxyl points away from the binding site. This indicates that it might be a less accessible interaction partner for ligand binding than suggested by the original single-conformer model.

Thirdly, radicicol (RDC) is a potent macrocyclic inhibitor of HSP90-dependent tumor growth (Roe *et al.*, 1999 ▸). The overall fold between both the human and *C. albicans* Hsp90α structure (r.m.s.d.s of 1.4 Å for chain *A* and 1.2 Å for chain *B*) and RDC pose (r.m.s.d. of 0.14 Å for both chains) are similar. In both homologs, RDC forms hydrogen bonds with the conserved residue Asp93/82 [Fig. 3 ▸(*c*)]. However, our analysis revealed a second high-energy conformation of Asp93/82 in the *C. albicans* structure. Notably, in the human structure the dynamic Lys58/47 engages with RDC, while no direct interactions are formed in the *C. albicans* structure. Using *FLEXR-MSA* we detected a second weak conformation of this Lys in the electron-density maps of the human form that points away from RDC [Fig. 3 ▸(*d*)].

### 3.4. Detecting conformational differences in HSP90 across three homologs

Next, we expanded our comparison from human and *C. albicans* to a third homolog by considering HSP90 from the parasitic protist *Trypanosoma brucei* bound to SNX-2112. There is a considerable rearrangement of ‘helical’ residues Val93–Ser102 in the *T. brucei* and *C. albicans* forms bound to SNX-2112 that is absent in the human form (Whitesell *et al.*, 2019 ▸; Supplementary Fig. S3). These differences within the same lid state were proposed to contribute to the variability in affinities between homologs (Whitesell *et al.*, 2019 ▸). Our analysis detected additional homolog-specific states away from the lid site that might allosterically modulate affinity. Specifically, we identified that the ligand-responsive Lys58/47 in RDC structures [Figs. 3 ▸(*d*)] also shifts conformation between homologs on binding SNX-2112 [Fig. 3 ▸(*e*)]. In both chains of the human structure, this Lys is in a consistent position and hydrogen-bonds to SNX-2112. The *C. albicans* structure contains two protein copies but only one chain is occupied by the ligand. The Lys in the bound chain is in a different conformation than in the human protein but remains hydrogen-bonded to the ligand, which shifts by an r.m.s.d. of 2.4 Å between the human and *C. albicans* structures. In contrast to the bound chain, this Lys points away from the binding site in the apo *C. albicans* chain. Interestingly, in the *T. brucei* structure the Lys is in a mixture of both the bound and unbound conformations from the *C. albicans* structure and results in a different ligand pose. The ring featuring the oxygen closest to the Lys is most affected (r.m.s.d.s of 2.8 Å to the human structure and 2.5 Å to that from *C. albicans*).

### 3.5. Mapping global, homolog-specific conformational differences

**Figure 4:**
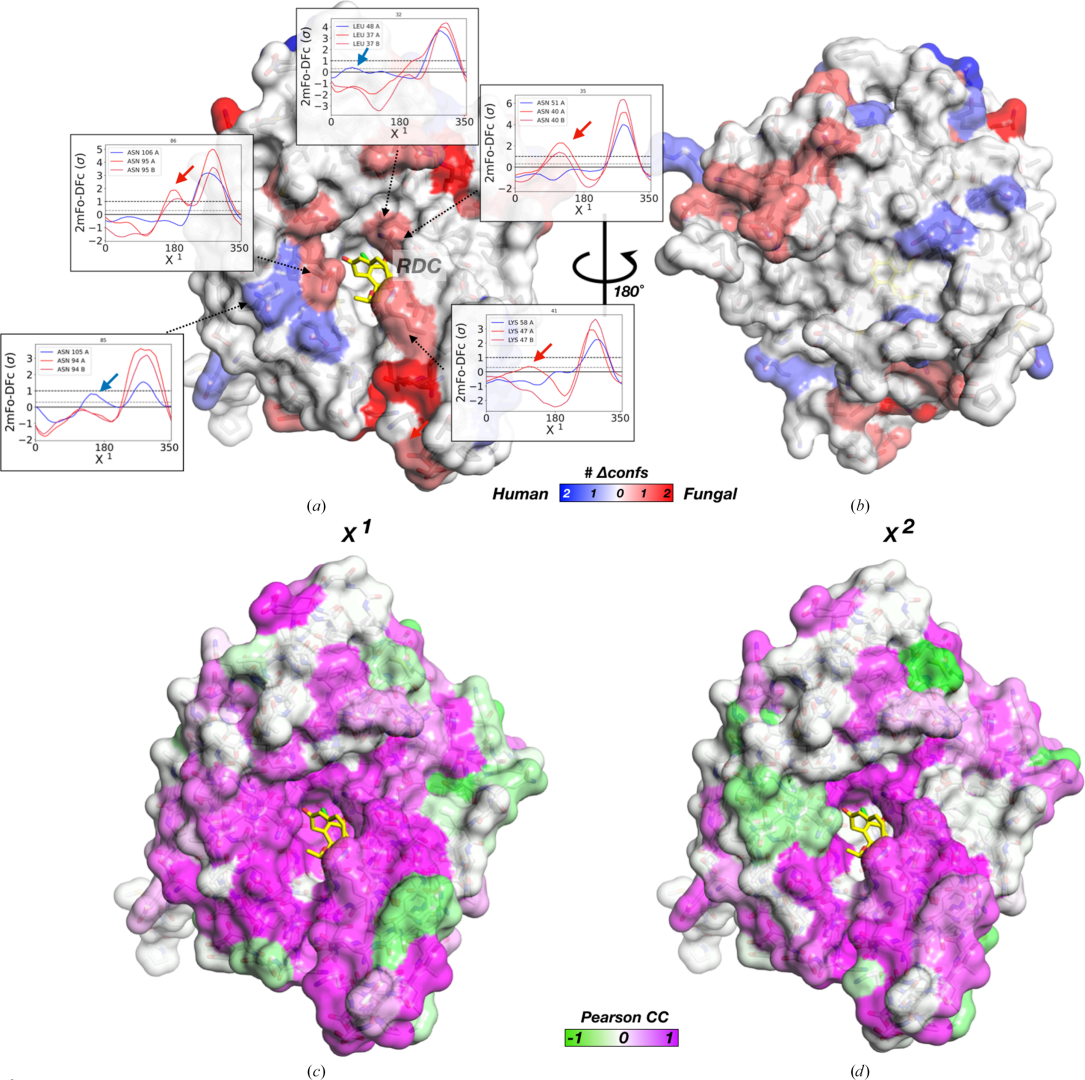
Mapping homolog-specific regional conformational variability in HSP90 bound to RDC. Difference in χ^1^*Ringer* peaks between human and *C. albicans* Hsp90α–RDC mapped onto the front (*a*) and back (*b*) of human Hsp90α–RDC. The red surface coloring corresponds to more alternative conformers as peaks in electron-density maps in the *C. albicans* protein and the blue surface corresponds to more peaks in human Hsp90α bound to RDC (shown as yellow sticks). *Ringer* plots are shown for binding-site residues with alternative conformations between homologs. This is also supported by mapping Pearson CC values for χ^1^ (*c*) and χ^2^ (*d*) onto the protein surface.

To quantify how many side-chain conformations change between human and *C. albicans* Hsp90α–RDC we subtracted the number of peaks in aligned *Ringer* plots [Figs. 4 ▸(*a*) and 4 ▸(*b*)]. Mapping these peak-count differences onto the protein surface allowed us to pinpoint local conformational differences in human and *C. albicans* RDC-bound structures. This revealed that several conserved residues in the RDC binding site in *C. albicans* HSP90 have additional conformations that are not present in the human structure. In contrast, flexibility in human HSP90 is greater for residues along the lid. While these residues do not directly interact with RDC, they provide ligand access to the pocket and often reposition upon binding ligands of different chemotypes (Stachowski & Fischer, 2022 ▸) [Figs. 4 ▸(*a*) and 4 ▸(*b*)]. Pearson CC values between aligned *Ringer* plots support this as well: while the canonical nucleotide-binding site is positioned similarly between homologs, the ATP lid is variable [Figs. 4 ▸(*c*) and 4 ▸(*d*), Supplementary Fig. S3]. Mapping these values across the entire protein surface shows additional regions with varying dynamics both near and far from the orthosteric binding site.

## 4. Discussion

At sufficient resolution, electron-density maps often contain details that describe protein dynamics that are missing in the deposited structural models. To enable an unbiased comparison of hidden, low-occupancy features in electron-density maps across diverse proteins, we combined electron-density sampling with MSA. We used this tool, *FLEXR-MSA*, to compare alternative side-chain conformations in electron-density maps of HSP90 across four human isoforms, a fungal homolog and a protist homolog.

Three main implications for ligand binding emerge from this work. Firstly, *FLEXR-MSA* revealed changes in the binding-site conformations of four human HSP90 isoforms, beyond obvious sequence dissimilarities, that were hiding in the electron-density maps. Secondly, we found homolog-specific conformational variability of charged residues in comparisons of three Hsp90α homologs bound to varying ligands. This was most profound for a conserved Lys that changed conformation with ligand pose between homologs and ligand-bound states. Thirdly, a protein-wide comparison of human and *C. albicans* Hsp90α bound to RDC showed different orthosteric and potential allosteric regions of heightened variability between homologs.

It is worth keeping in mind that even near-identical proteins differ in their conformational landscape. For instance, binding-site conformations are often connected to water networks, so that repopulating side chains will shift water networks and vice versa (Darby *et al.*, 2019 ▸). Likewise, differences in the amino-acid sequence alter water-network connectivity even if waters within the network are conserved. Taking advantage of this phenomenon to displace specific waters in HSP90 has provided an interesting route to selectively target the α and β isoforms (Khandelwal *et al.*, 2018 ▸; Mishra *et al.*, 2021 ▸; Huck *et al.*, 2019 ▸). Here, we observed additional weak conformations of a conserved Asp (Asp88) in two of the four Hsp90β chains. This change in populations might be an underappreciated consequence of the change from an adjacent Ala to Ser between α and β. In recent work we showed that waters in HSP90 isoforms bound to the same ligand, 6DMP, exhibited distinct behaviors regarding r.m.s.d. and normalized *B* factors (Stachowski *et al.*, 2023 ▸). This included waters that bridge interactions between 6DMP and this conserved Asp. In another example, changed lid states in Hsp90α bound to AUY-922 between the human and *C. albicans* proteins cascade through the binding site and reposition ligands, water networks and side chains. Connecting weakly populated states observed here with changes in water behavior and sequence differences might provide new insights to selectively target HSP90 homologs.

When trying to understand homolog-specific differences, the focus is generally on sequence differences. Here, we have illustrated the ability of *FLEXR-MSA* to detect homolog-selective repositioning of two conserved charged residues Lys (58 in α) and Asp (93 in α) in the nucleotide binding site. For instance, in the case of *C. albicans* Hsp90α bound to SNX-2112 the Lys exists in two distinct conformations between ligand-bound and unbound states. Consequently, the ligand moiety interacting with the Lys also varied with the Lys conformation while the rest of the pose was conserved across homologs. This same Lys in the human homologue was observed to be temperature-sensitive (Stachowski *et al.*, 2022 ▸) and a selective handle for covalent inhibitor design (Cuesta *et al.*, 2020 ▸). Whitesell and coworkers reported that SNX-2112 exhibited a threefold higher affinity for *C. albicans* Hsp90α over human (Whitesell *et al.*, 2019 ▸) and others reported a higher affinity for the *T. brucei* protein over human (Pizarro *et al.*, 2013 ▸). AUY-922 also exhibited higher affinity for the human form compared with that from *C. albicans* (Whitesell *et al.*, 2019 ▸). Hidden changes in homolog-specific flexibilities might explain some of these differences in affinities.

While the *FLEXR-MSA* approach facilitates the inspection of electron-density maps for alternative side-chain conformations in sequence-diverse proteins, the responsibility for sensible data input and cautious analysis is still with the user (Pozharski *et al.*, 2013 ▸). Users need to carefully consider other influences on structure such as space group, resolution and crystallization and experimental conditions. For instance, to test the consistency of these observations we treated chains as separate lines of evidence when possible. Differences in conformations between chains could result from varying microenvironments within the crystal lattice. However, with careful consideration that specific rotamers are not distorted involuntarily, the presence of conformational heterogeneity alone can be enlightening. Also, differences in resolution will create different thresholds for detecting high-energy, weakly populated states. For instance, the additional Asp conformations in β (1.8 Å) were not present in Grp94 (2.3 Å) or Trap1 (2.3 Å) although all three share the adjacent Ala substitution in lieu of Ser in α. We cannot rule out that rare Asp conformations are absent due to the reduced signal-to-noise ratio at the lower resolution of the Grp94 and Trap1 structures. The best way to validate these observations is to add the conformations into the model, for example with *FLEXR*, re-refine the structure against the diffraction data and monitor occupancies and clashes (Stachowski & Fischer, 2023 ▸, 2024 ▸).

Also, the robustness of the *FLEXR-MSA* approach is directly dependent on the success of the sequence alignment, which in turn is linked to sequence similarity and the completeness of sequences as they are extracted from the *Ringer* output, which excludes residues without χ angles, such as Ala and Gly, or unbuilt portions of the model. Inherently sequence alignments can be quite poor at the beginning and end of chains and adjacent to unbuilt loops. However, these regions also typically correspond to areas of weak electron density and this lack of signal reduces confidence in any observation in that region of the protein so that analysis may not be useful. To overcome this potential limitation, *FLEXR-MSA* saves alignment and re-indexing files that can be referenced and modified. If the alignment approach appears to be limiting, *FLEXR-MSA* allows the users to manually change the alignment and *MUSCLE* contains several options that can additionally be adjusted to improve the alignment (Edgar, 2004 ▸).

*FLEXR-MSA* was designed for comparing electron densities of structures with mutations, dissimilar sequences and misnumbered residues. Combing electron-density sampling with MSA bypasses many tedious steps and allows users to quickly visualize and analyze electron-density features of structures with non-identical sequences. *FLEXR-MSA* is fast, portable and relies only on common dependencies. It is available within the *FLEXR* suite, and, as such, is freely available to the community. 

## Supplementary Material

Supplementary tables and figures, and supplementary methods. DOI: 10.1107/S2052252525001332/jt5079sup1.pdf
