# Supplementary material for: FLEXR-MSA: electron-density map comparisons of sequence-diverse structures
Source: IUCrJ. 2025 Feb 27;12(Pt 2):245–54. doi: 10.1107/S2052252525001332 (PMC11878447; doi:10.1107/S2052252525001332)
Supplement: Supplementary file 1 [file m-12-00245-sup1.pdf]

IUCrJ

Volume 12 (2025)

Supporting information for article:

FLEXR-MSA: electron-density map comparisons of sequence-diverse structures

Timothy R. Stachowski and Marcus Fischer

```
Trap1     TSKHEFQAETKKLLDIVARSLYSEKEVFIRELISNASDALEKLRHKLVSDGQALP---EM     57
Grp94     SEKFAFQAEVNRMMKLIINSLYKNKEIFLRELISNASDALDKIRLISLTDENALSGNEEL     60
Hsp90a    VETFAFQAEIAQLMSLIINTFYSNKEIFLRELISNSSDALDKIRYETLTDPSKLDSGKEL     60
Hsp90b    VETFAFQAEIAQLMSLIINTFYSNKEIFLRELISNASDALDKIRYESLTDPSKLDSGKEL     60
           ... ****  :::.:: .::*.:**:*:******:****:*:*   ::* . *    *:

Trap1     EIHLQTNAEKGTITIQDTGIGMTQEELVSNLGTIARSGSKAFLDALQNQA---EASSKII     114
Grp94     TVKIKCDKEKNLLHVTDTGVGMTREELVKNLGTIAKSGTSEFLNKMTEAQEDGQSTSELI     120
Hsp90a    HINLIPNKQDRTLTIVDTGIGMTKADLINNLGTIAKSGTKAFMEALQAG-----ADISMI     115
Hsp90b    KIDIIPNPQERTLTLVDTGIGMTKADLINNLGTIAKSGTKAFMEALQAG-----ADISMI     115
           :.:  : :.  : : ***:***: :*:.******:**:. *:: :        :  .:*

Trap1     GQFGVGFYSAFMVADRVEVYSRSAAPGSLGYQWLSDGSGVFEIAEASG--VRTGTKIIIH     172
Grp94     GQFGVGFYSAFLVADKVIVTSKHN--NDTQHIWESDSNEFSVIADPRGNTLGRGTTITLV     178
Hsp90a    GQFGVGFYSAYLVAEKVTVITKHN--DDEQYAWESSAGGSFTVRTDTGEPMGRGTKVILH     173
Hsp90b    GQFGVGFYSAYLVAEKVVVITKHN--DDEQYAWESSAGGSFTVRADHGEPIGRGTKVILH     173
          **********::**::* * ::    ..  : * *...    :    *  :  **.: :

Trap1     LKSDCKEFSSEARVRDVVTKYSNFVSFP     200
Grp94     LKEEASDYLELDTIKNLVKKYSQFINFP     206
Hsp90a    LKEDQTEYLEERRIKEIVKKHSQFIGYP     201
Hsp90b    LKEDQTEYLEERRVKEVVKKHSQFIGYP     201
          **.: .:: .   ::::*.*:*:*:.:*
```

Figure S1 Sequence alignment of human HSP90 isoforms. Binding site residues are bolded, and non-conserved binding site residues are highlighted in yellow. The isoform coloring follows the same color scheme used for Fig. 2.

Table S1 PDB codes, space groups, and resolutions for human HSP90-6DMP structures.

**Property**	**Isoform**			
	**Hsp90α**	**Hsp90β**	**Grp94**	**Trap1**
**PDB code**	4FCP	7ULJ	7ULL	7ULK
**Resolution (Å)**	2.0	1.8	2.4	2.3
**Space group**	P21212	P65	P21212	P1211

```
H. sapiens  -VETFAFQAEIAQLMSLIINTFYSNKEIFLRELISNSSDALDKIRYESLTDPSKLDSGKE 59
C. albicans -GETHEFTAEISQLMSLIINTVYSNKEIFLRELISNASDALDKIRYQALSDPSQLESEPE 59
T. brucei   MTETFAFQAEINQLMSLIINTFYSNKEIFLRELISNSSDACDKIRYQSLTNQ----SVPH 56
              **. * *** *********.**************:*** *****::*::     *  .

H. sapiens  LHINLIPNKQDRTLTIVDTGIGMTKADLINNLGTIAKSGTKAFMEALQAGADISMIGQFG 119
C. albicans LFIRIIPQKDQKVLEIRDSGIGMTKADLVNNLGTIAKSGTKSFMEALSAGADVSMIGQFG 119
T. brucei   LRIRVIPDRVNKTLTVEDSGIGMTKADLVNNLGTIARSGTKSFMEALEAGGDMSMIGQFG 116
            * *.:**:: ::.* : *:*********:*******:****:*****.**.*:*******

H. sapiens  VGFYSAYLVAEKVTVITKHNDDEQYAWESSAGGSFTVRTD-TGEPMGRGTKVILHLKEDQ 178
C. albicans VGFYSLFLVADHVQVISKHNDDEQYVWESNAGGKFTVTLDETNERLGRGTMLRLFLKEDQ 179
T. brucei   VGFYSAYLVADRVTVVSKNNEDDAYTWESSAGGTFTVTST-PDCDLKRGTRIVLHLKEDQ 175
            ***** :***::* *::*:*:*: *.***.***.***     .  : *** : *.*****

H. sapiens  TEYLEERRIKEIVKKHS-FIGYPITLFVEK---- 207
C. albicans LEYLEEKRIKEVVKKHSEFVAYPIQLVVTKEVEK 213
T. brucei   QEYLEERRLKDLIKKHS---GYDIELMVEN---- 202
             *****:*:*::****   .* * *.* :
```

Figure S2 Sequence alignment of Hsp90α NTD homologs**.** Binding site residues are bolded, non-conserved binding site residues are highlighted in yellow. The human (blue) sequence shares 72% identity with *C. albicans* (red) and 71% with *T. brucei* (purple). *T. brucei* and *C. albicans* sequences share 69% identity. The isoform coloring follows the same color scheme used for Fig. 3.

Table S2 PDB codes, space groups, and resolutions for human, *C. albicans*, and *T. brucei* structures.

**Property**	**Homolog**		
**Apo**	**Human**	***C. albicans***	
**PDB code**	1UYL	6CJI	
**Resolution (Å)**	1.4	1.6	
**Space group**	I222	P4322	
**SNX-2112**	**Human**	***C. albicans***	***T. brucei***
**PDB code**	4NH7 (chains A and B)	6CJR (chains A and B)	3OPD (chain A)
**Resolution (Å)**	2.0	1.8	2.6
**Space group**	C121	P43	P212121
**AUY-922**	**Human**	***C. albicans***	
**PDB code**	2VCI	6CJS	
**Resolution (Å)**	2.0	1.9	
**Space group**	I222	I4122	
**RDC**	**Human**	***C. albicans***	
**PDB code**	4EGK	6CJL (chains A and B)	
**Resolution (Å)**	1.7	1.7	
**Space group**	P1211	P43	

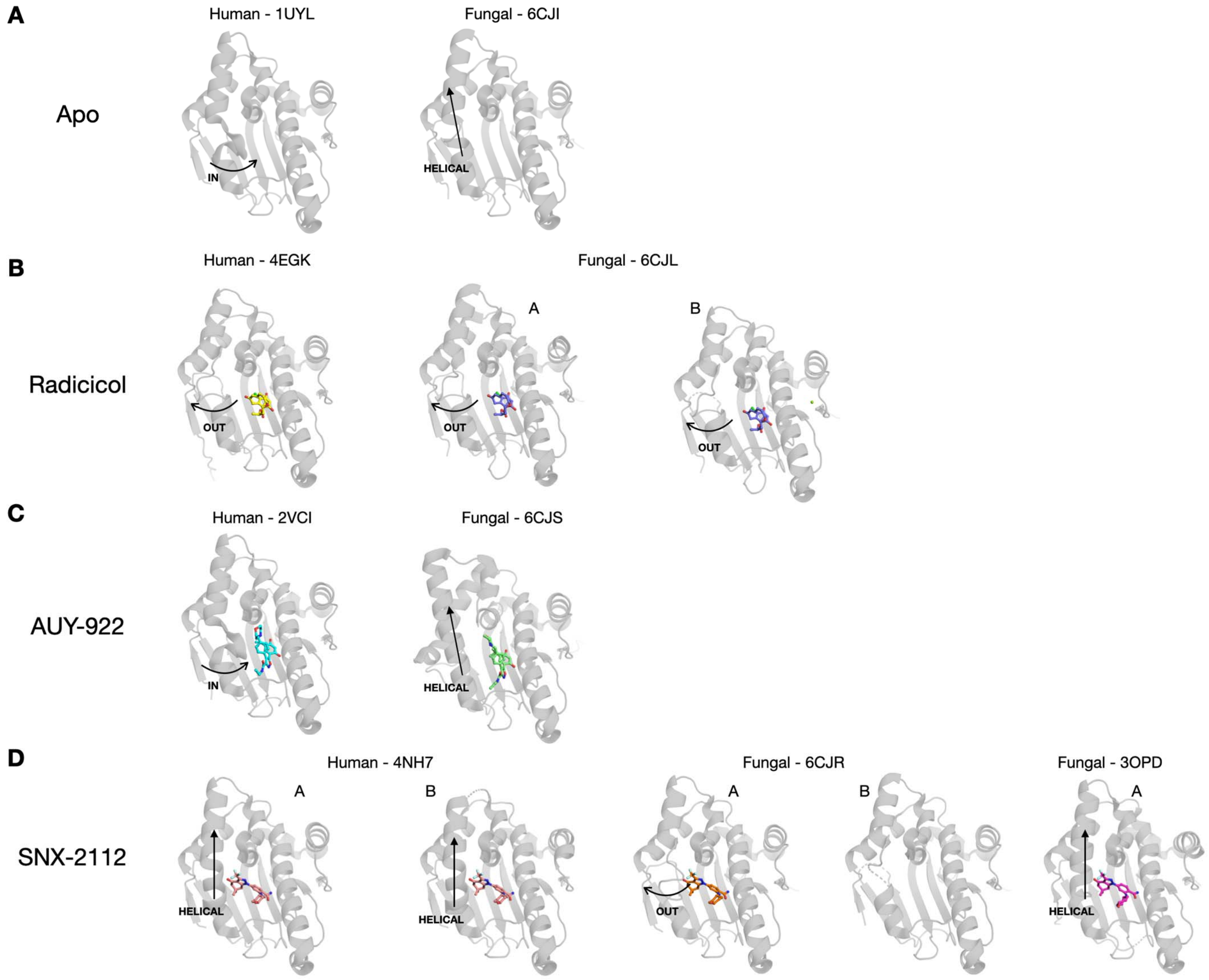

Figure S3 Overview of ATP Lid conformations across (A) apo structures and bound to (B) radicicol, (C) AUY-922, and (D) SNX-2112.

S1. Supplementary Methods

Full *FLEXR* functionality requires Coot 1.1.10, which we recommend installing through CCP4 9 (https://www.ccp4.ac.uk/download/). Details are available on GitHub: https://github.com/TheFischerLab/FLEXR. *FLEXR-MSA* was written in Python 3.9 on macOS 14.7 with an Apple silicon processor. *FLEXR* requires Biopython, Matplotlib, Numpy, Pandas, and SciPy packages. These can be installed within an Anaconda environment or separately using pip:

```
pip3 install numpy
```

MUSCLE v5.2 (http://www.drive5.com/muscle/) can be installed with Homebrew:

```
brew install muscle
```

Ringer (is best accessed through the mmtbx library (https://cctbx.github.io/mmtbx/mmtbx.html), which comes packaged with Phenix. *Ringer* measurements can be calculated with:

```
mmtbx.ringer somepdb.pdb somepdb_map_coeffs.mtz
```

FLEXR-MSA can be downloaded by cloning the GitHub repository:

```
git clone https://github.com/TheFischerLab/FLEXR.git
```

FLEXR-MSA works from the standard *Ringer* output CSV files and is executed through the command line. Users the colors corresponding to each file, using command line options `colors`:

```
python flexr.py -m MSA -colors blue,red,crimson
```

If no colors are defined random ones are assigned. The program starts by alphabetically loading all *Ringer* outputs with the `_ringer.csv` extension in the working directory and prints a legend with files corresponding to the chosen colors. The color log is saved to `plot_legend.txt`

```
Data Color
4EGK_A_ringer.csv blue
6CJL_A_ringer.csv red
6CJL_B_ringer.csv crimson
```

The program first extracts the amino acid sequence for each file and formats them into FASTA format and exports it to a single file:

```
ringer_alignment.fasta
```

MUSCLE is used to perform a multiple sequence alignment algorithm. The aligned sequences are saved to:

```
ringer_alignment_muscle.fasta
```

The aligned sequences are extracted and converted into a Pandas dataframe. Each position in the alignment (including gaps, -) is assigned a new index value that relates aligned residues ("res" column). This table is saved to `alignment_new_index.csv` and looks like this:

4EGK_A_ringer.csv	6CJL_A_ringer.csv	6CJL_B_ringer.csv	res
P	-	-	0
M	-	-	1
E	-	-	2
E	E	E	3
V	T	T	4
E	H	H	5
T	-	E	6
F	F	F	7
F	T	T	8

The program returns to the original *Ringer* output CSV files and adds a column with the new index values for each residue. The updated *Ringer* CSV files are merged into a single dataframe. The new index values are looped over and data for residues with corresponding index values are called for plotting. The plotting portion creates a directory corresponding to the input chi value (e.g. `./chi1`) where the plots are saved. Plot titles and file names are labeled with the index value. The plot legends contain the original PDB residue ID followed by the chain ID. An example file is shown here. The title of the file is `37chi1.png` corresponding the 37th aligned position measured at Chi^1:

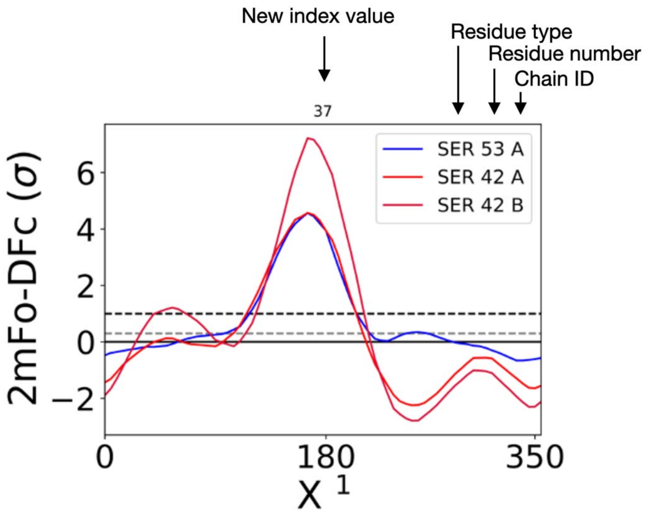

Side-chains with branched torsion angles, such as the first dihedral of Val, always yield a minimum of two peaks in *Ringer* plots. In the case where a branched residue is substituted in another structure with a non-branched residue (such as Val to Leu) can mistakenly be interpreted as a change in the number of conformations at that position between the two structures. The default option in *FLEXR-MSA* is to avoid

this situation and only compare residues with unbranched torsion angles. The user can override this with `-safety False` so that all aligned residues are compared.

In some situations, parts of sequences can be poorly aligned and will produce erroneous comparisons. This can be due to poor sequence similarity at the beginning and end of sequences or near unmodeled portions of models like loops. Users can manually adjust the re-indexed alignment file, `alignment_new_index.csv`, and rerun *FLEXR-MSA* by setting `-reload True` .

FLEXR-MSA can also calculate Pearson correlation coeffecients pairwise between *Ringer* plots of each residue by setting the `-pearson` option to `True`. This will produce heatmaps reflecting pairwise CC values in the directory `chi`*n*`_cc` and a CSV files of values: `cc_chi`*n*`.csv`.

Finally, setting the `-render` option to `True` will map the median CC values to the B-factor column in the first input PDB file (if present in the working directory). This allows the user to visualize the values on the protein with a program like PyMOL, which was done to produce Figure 4.
